# Risk factors for cardiometabolic health in Ghana: Cardiometabolic Risks Study Protocol-APTI Project

**DOI:** 10.3389/fendo.2024.1337895

**Published:** 2024-09-04

**Authors:** Thomas Hormenu, Iddrisu Salifu, Ebenezer Oduro Antiri, Juliet Elikem Paku, Aaron Rudolf Arthur, Benjamin Nyane, Eric Awlime Ableh, Augustine Mac-Hubert Gablah, Cecil Banson, Samuel Amoah, Marie Consolatrice Sage Ishimwe, Regine Mugeni

**Affiliations:** ^1^ Department of Health, Physical Education and Recreation, University of Cape Coast, Cape Coast, Ghana; ^2^ Cardiometabolic Epidemiology Research Laboratory, University of Cape Coast, Cape Coast, Ghana; ^3^ Centre for Coastal Management-Africa Centre of Excellence in Coastal Management, Cape Coast, Ghana; ^4^ Directorate of University Hospital, University of Cape Coast, Cape Coast, Ghana; ^5^ Institute of Global Health Equity, University of Global Health Equity, Butaro, Rwanda; ^6^ Kibagabaga Level Two Teaching Hospital, Rwanda Ministry of Health, Kigali, Rwanda

**Keywords:** undiagnosed diabetes, prediabetes, hypertension, lifestyle intervention, Ghana, OGTT, protocol

## Abstract

**Introduction:**

Cardiometabolic diseases are rapidly becoming primary causes of death in developing countries, including Ghana. However, risk factors for these diseases, including obesity phenotype, and availability of cost-effective diagnostic criteria are poorly documented in an African-ancestry populations in their native locations. The extent to which the environment, occupation, geography, stress, and sleep habits contribute to the development of Cardiometabolic disorders should be examined.

**Purpose:**

The overall goal of this study is to determine the prevalence of undiagnosed diabetes, prediabetes, and associated cardiovascular risks using a multi-sampled oral glucose tolerance test. The study will also investigate the phenotype and ocular characteristics of diabetes and prediabetes subgroups, as well as determine if lifestyle changes over a one-year period will impact the progression of diabetes and prediabetes.

**Methods and analysis:**

The study employs a community-based quasi-experimental design, making use of pre- and post-intervention data, as well as a questionnaire survey of 1200 individuals residing in the Cape Coast metropolis to ascertain the prevalence and risk factors for undiagnosed diabetes and prediabetes. Physical activity, dietary habits, stress levels, sleep patterns, body image perception, and demographic characteristics will be assessed. Glucose dysregulation will be detected using oral glucose tolerance test, fasting plasma glucose, and glycated hemoglobin. Liver and kidney function will also be assessed. Diabetes and prediabetes will be classified using the American Diabetes Association criteria. Descriptive statistics, including percentages, will be used to determine the prevalence of undiagnosed diabetes and cardiovascular risks. Inferential statistics, including ANOVA, t-tests, chi-square tests, ROC curves, logistic regression, and linear mixed model regression will be used to analyze the phenotypic variations in the population, ocular characteristics, glycemic levels, sensitivity levels of diagnostic tests, etiological cause of diabetes in the population, and effects of lifestyle modifications, respectively. Additionally, t-tests will be used to assess changes in glucose regulation biomarkers after lifestyle modifications.

**Ethics and dissemination:**

Ethics approval was granted by the Institutional Review Board of the University of Cape Coast, Ghana (UCCIRB/EXT/2022/27). The findings will be disseminated in community workshops, online learning platforms, academic conferences and submitted to peer-reviewed journals for publication.

## Introduction

Research has established that obesity, high levels of glucose, and triglycerides are significant contributors to the risks of developing cardiometabolic diseases [CMDs] (1–4). However, literature suggests that two-thirds of African-born individuals in sub-Saharan Africa living in America diagnosed with abnormal glucose tolerance are more likely to be non-obese (5). This has led to research to establish the specific causes of diabetes and prediabetes in an African population, and to identify risk factors unique to Africans. The main mechanisms for the development of diabetes are beta-cell failure and insulin resistance, with abnormal glucose tolerance representing an imbalance between the degree of insulin resistance and the β-cell secretion (6–8). Identifying the etiology of diabetes is critical for prescribing the relevant treatment or interventions (6).

Globally, 537 million adults live with diabetes, with Africa contributing 4.47% (24 million) and witnessing 416,000 diabetes-related deaths in 2021 alone (9). According to the International Diabetes Federation (9), this prevalence is projected to double by 2045 due to high rates of abnormal glucose tolerance, including prediabetes (5, 10–12). Prediabetes and diabetes represent a glycemic continuum with both associated with vascular complications and an increased burden on all-cause mortality rates. Presently, 179.2 million individuals globally with undiagnosed diabetes (13), with the IDF Africa Region standing out with the highest rate of undiagnosed cases among all IDF regions, at 60% (14). This underscores the critical need for increased awareness, early detection, and intervention strategies to address undiagnosed diabetes in Africa.

Many pathways contribute to abnormal glucose tolerance, and predisposing factors can differ among populations (8, 15). Although there is data on diabetes in populations of African descent, such as African Americans and African-Caribbean’s (15),, these data cannot be used to infer about African-born individuals in the sub-Saharan Africa. There have been divergent views on the major contributor to abnormal glucose intolerance, with a study (7) proposing beta-cell failure, and other studies (6, 8) implicating insulin resistance. This highlights the importance of conducting studies within diverse Sub-Saharan African populations to identify population-specific risk factors. Additionally, with increasing physical inactivity, consumption of high energy-dense foods, social alcohol drinking, and the sedentary lifestyle of Africans living in Africa (16), assessing risks for cardiometabolic diseases and promptly provide health promotion interventions is relevant.

Diabetes poses a significant challenge to global public health and its burden doubled with the emergence of the COVID-19 pandemic. In Africa, most COVID-19 deaths were significantly associated with underlying health conditions, including diabetes and other cardiovascular disease risks. A systematic review established that diabetes mellitus was associated with higher COVID-19 mortality rates in sub-Saharan Africa (17). The findings indicated a statistically significant association between diabetes mellitus and increased mortality among COVID-19 inpatients, showing a 1.39-fold higher risk of death in patients with diabetes mellitus compared to those without.

Notwithstanding, there is still a dearth of accurate and revealing data on the prevalence of diabetes and other cardiovascular and metabolic disease (CMD) risks in sub-Saharan Africa. This study seeks to establish knowledge about the prevalence of diabetes and other CMD risks, to reduce diabetes prevalence and prepare for potential future pandemics. More than 75% of the countries in sub-Saharan Africa lack contemporary primary data on diabetes prevalence (18), with estimates in the sub-region relying on data extrapolation and mathematical modeling. However, consolidating data from the various countries within sub-Saharan Africa would help facilitate the collection of more comprehensive information regarding the etiology and presentation of abnormal glucose tolerance in the sub-region. By doing so, Africa can gain a more accurate understanding of its diabetes burden and risk factors, enabling proactive measures to reverse the trajectories of the diabetes epidemic projected by the IDF for 2045 through early detection and diagnosis.

Early diagnosis of diabetes is crucial for mitigating its debilitating effects, including peripheral neuropathy, retinopathy, diabetic foot complications, and the susceptibility to infectious diseases (17, 19, 20). The estimated prevalence of diabetes in Ghana is 6.5% (21), suggesting that one in every 15 Ghanaians has diabetes, signifying the onset of a worrying epidemic. Diabetes is a major contributor to mortality in Ghana, with 9,778 deaths reported in 2017, up from the 8528 in 2014 (22). The cost of treating one person with diabetes in Ghana as at 2017 was a high $106.5. Diabetes is reaching epidemic proportions in sub-Saharan Africa, with the region experiencing the most rapid increase in type 2 diabetes (T2D) worldwide and the highest proportion undiagnosed diabetes (9). To address this epidemic and its complications, comorbidities and mortalities, measures must encourage the early diagnosis of diabetes and reduce undiagnosed diabetes (9). Identifying and developing African-specific tools and interventions to improve the early diagnosis of diabetes and prescribe effective treatments requires more clinical research in the African population. The prospective findings of this research should be incorporated into the clinical care of prediabetes and diabetes, and replicated across many African countries in both rural and urban areas. Given that Ghana has a typical African population and an epidemic-like prevalence of diabetes (21), data from this study can bridge developing and developed countries, increasing the degree of certainty of data extrapolation and modeling of diabetes-related prevalence and outcomes.

The increased susceptibility to diabetes in people from lower- and middle-income countries has been well explored (9, 23–25). In Ghana, the extent to which obesity phenotypes, dietary habits, socioeconomic status, perceived stress, sleep patterns, geographical location, and occupational characteristics contribute to cardiometabolic risk development has not been well explored (26–30), especially due to diagnostics and methodological limitations. There is still a dearth of data concerning the actual prevalence of undiagnosed diabetes in Ghana, the specific interventions to remedy these risk factors, and how culturally sensitive non-pharmacological interventions such as diet and physical activity can contribute to diabetes remission in newly diagnosed persons with abnormal glucose tolerance.

Non-African studies have shown the possibility of diabetes and prediabetes remission through non-pharmacological interventions (31, 32), making it important to replicate and improve on these studies in a typical African-born population. Although some studies from developed countries have shown lifestyle changes to significantly influence diabetes remission (33–36), no prospective study done in sub-Saharan Africa and Ghana has shown the possibility of people newly diagnosed with diabetes reverting to prediabetes or people with prediabetes reverting to normal glucose tolerance through non-pharmacological lifestyle interventions. These reasons, as well as the high prevalence of undiagnosed diabetes and the projected increase of 143% by 2045, have necessitated the inception and execution of this project. This project assesses the role of lifestyle choices, environmental exposures, psychosocial factors and nutrition on the prevalence of obesity, hypertension and diabetes (CMD risk factors) among Ghanaians, and how these contribute to improved health and visual outcomes.

**Figure 1:**
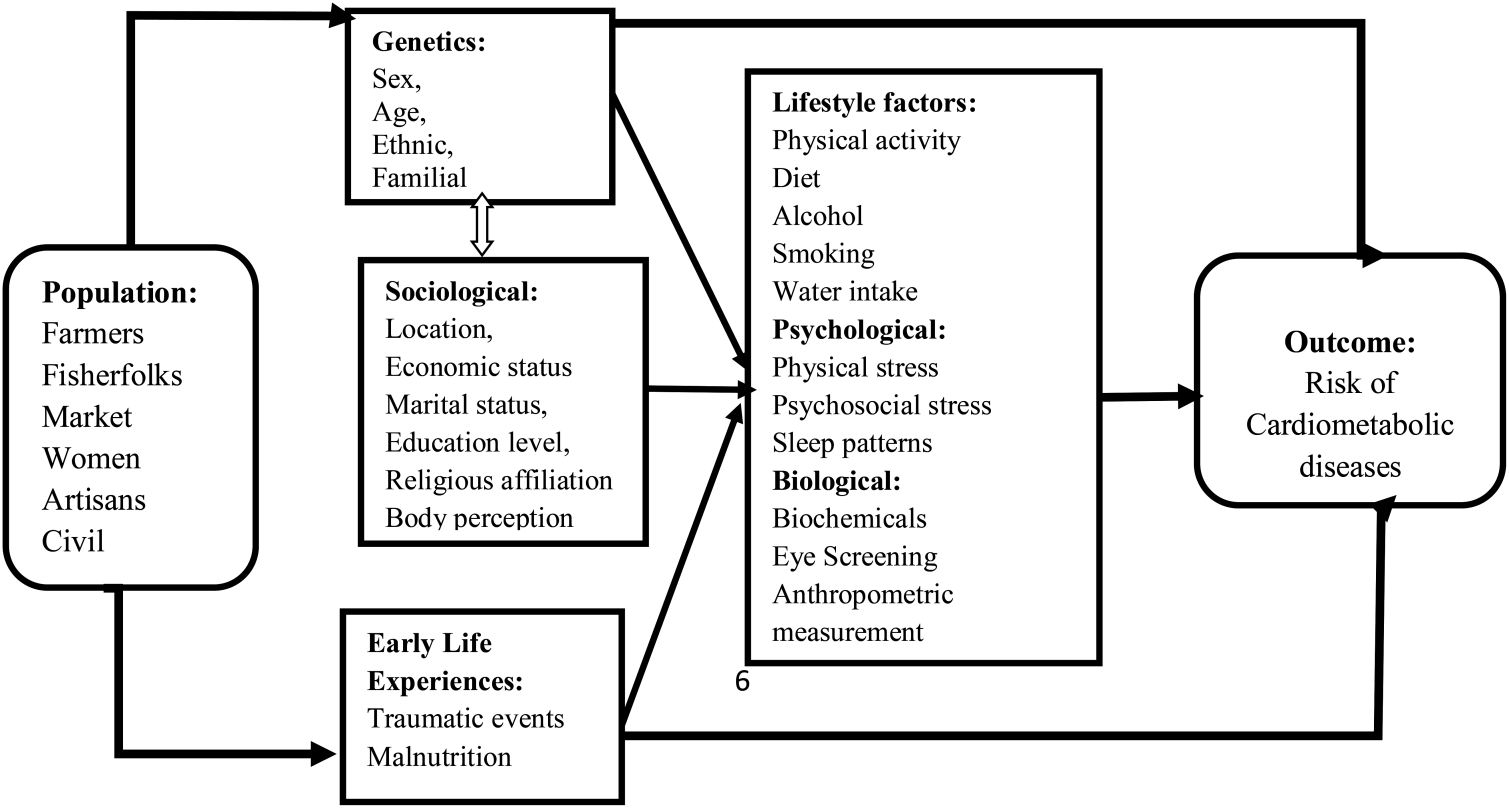
A conceptual framework for the CarMeR Study.

Due to the demographic characteristics and nature of diabetes, the biopsychosocial model of disease development underpins the CarMeR Study. The model as utilized in a previous study, propounds that disease occurrence results from the interplay of biological, psychological and social factors (37). It explains how factors, including biological factors (family history diabetes, sex), sociological factors (familial, occupation, economic status, nutrition, ethnicity, place of birth and early life experiences), and psychological factors (stress and sleep patterns) contribute to disease development. The biopsychosocial model suggests that each of these three domains has an influence on health. The biopsychosocial model has been selected because it provides a framework for understanding risk factors associated with CMDs in Ghana. As illustrated in **Figure 1**, we hypothesize that genetic factors, social factors, familial factors, stress, economic status, religion, ethnicity, nutrition patterns, physical activity and other population characteristics play a crucial role in determining an individual’s susceptibility to CMDs. Incorporating these factors into our conceptual model, we envisage gaining a deeper understanding of the complex interactions that contribute to the risks of CMDs such as diabetes, obesity, hypertension, and other cardiovascular diseases. This model will explore the prevalence of undiagnosed diabetes, prediabetes and hypertension among critical populations in Ghana, and how various factors predispose individuals to risk of cardiometabolic diseases.

This project, dubbed the Cardiometabolic Risks (CarMeR) Study, is by the Bill and Melinda Gates Foundation through the African Postdoctoral Initiative, managed by the African Academy of Sciences. The objectives are: 1. determine the prevalence of undiagnosed diabetes, prediabetes and other associated cardiovascular disease risks using the multi-sampled oral glucose tolerance test [OGTT] with 82.5g monohydrate dextrose, 2. characterize the phenotype of diabetes and prediabetes relative to body composition, body mass index (BMI), and other anthropometric measures, 3. establish the ocular characteristics and complications in people with diabetes and prediabetes, 4. ascertain whether the etiology of undiagnosed diabetes is predominantly insulin resistance or beta-cell insufficiency using homeostasis model analysis of beta [HOMA-β and HOMA-IR], 5. determine the sensitivity of diagnostic alternatives to the multi-sampled OGTT, specifically the fasting plasma glucose (FPG) test and the glycated hemoglobin (HbA1c) test, and 6. determine, over a 1-year period, if changes in food choices, physical activity, weight loss and psychosocial counseling influence reversal or prevention among people newly diagnosed with diabetes and prediabetes.

## Methods

The CarMeR Study will be executed by a multidisciplinary team of researchers from the University of Cape Coast, its Directorate of Health Services, the Rwanda National Hospital, and the University of Global Health Equity, Rwanda. To obtain reliable and valid results, the CarMeR Study will adhere to gold standards in all aspects of the project, including but not limited to the selection of communities, standardization of study protocols, adherence to ethical principles in the sampling procedures, anthropometric measurements, blood glucose testing, chemistry measurements, eye examination, blood pressure measurements, and surveys. The CarMeR Study will adopt the WHO-STEPwise approach for assessing cardiometabolic risk factors The STEPwise approach to surveillance (STEPS) is an NCD surveillance tool recommended by the WHO since 2005 for monitoring trends across countries (38, 39). The WHO-STEPwise instrument comprises three distinct phases or “steps” for evaluating risk factors. The first step is a questionnaire-based assessment, the second step comprises of physical measures and the third step comprises of biochemical measures (40). This instrument is a simplified and standardized approach to surveillance for collecting, analyzing and disseminating data on key risk factors especially for NCDs (38). In addition to a questionnaire survey, a community-based quasi-experimental design will be utilized to assess the glucose tolerance of all participants after administration of 82.5g monohydrate dextrose, in order to ascertain the prevalence and risk factors for undiagnosed diabetes and prediabetes. Subsequently, all consenting participants diagnosed with abnormal glucose tolerance will be enrolled into a community-based randomized controlled trial (RCT) to measure the effectiveness of lifestyle interventions on diabetes remission.

### Population

The population comprises adults aged between 25 and 70 years living in households within the Cape Coast metropolis. The choice of this age range was influenced by the fact that they are at a higher risk of developing diabetes in the metropolis (41, 42). It is estimated that the number of people between the ages of 25 and 70 living in the Cape Coast Metropolis is 102,533 (43), spread across several households and communities in the metropolis. The sample for the study is being drawn from rural, peri-urban, and urban communities in the Cape Coast Metropolis. Owing to the heterogeneous nature of the Cape Coast population, the selection of participants for this study is based on different characteristics. These characteristics includes the type of community (rural, peri-urban, urban), main economic activity (farming, fishing, trading, formal work), dominant religion (Christianity, Islam, African traditional religion), and ethnicity (Fante, Ewe, Asante, northern tribes).

### Participants

Following the protocol of the Research on Obesity and Diabetes among African Migrants (RODAM) study, which focused on obesity and type 2 diabetes among African migrants (44), this study estimates the prevalence of undiagnosed type 2 diabetes (T2D) in the Cape Coast metropolis of Ghana, at 6-7%, and the prevalence of obesity at 17%. The study aims for a power of 0.90 with α=0.05, including the Bonferroni correction. Using these parameters, a sample size of approximately 1000 would be sufficient to detect a 10% proportion of undiagnosed T2D. In order to compensate for attrition, the study seeks to recruit 1200 self-identified healthy individuals from 13 urban and rural communities in the Cape Coast Metropolis, who have not been previously diagnosed of diabetes or prediabetes prior to the study for collection of baseline data.

Due to the large and geographically dispersed population, a multistage sampling technique would be employed to sample participants for this study. This will be done in three stages, where the first stage involves dividing the population into clusters, based on the already existing communities within the metropolis. The second stage involves sampling these clusters to be used in the study by using systematic random sampling. At this stage, 13 clusters representing 13 rural, peri-urban and urban communities would be selected for the subsequent sampling stage. Finally, the third stage would involve further sampling within each selected cluster to randomly select households, and recruit the first two persons from each family line (with random replacement) in the indigenous communities, while using convenience sampling in non-indigenous communities. It is envisaged that approximately 90-100 participants will be randomly selected from three rural communities (Efutu Mampong, and Efutu), four fishing communities (Abekam, Duakor, Ekon, and Ntsen), three peri-urban communities (Ayeko-Ayeko, Ankaful and Nkanfoa) and one urban community (UCC), two market communities (Kotokuraba and Abura Markets), and one artisanal community (Siwdu). Household mapping will be done utilizing the 2021 Housing Census Data. All the 1200 volunteers will be recruited from their communities of origin before disclosing any monetary benefits.

#### Inclusion criteria

Participants would be included in the study if they aged 25 to 70 years, and have not been diagnosed with prediabetes or diabetes. Additionally, participants should be willing to undergo the screening procedure (glucose testing, eye examination, BMI assessment, lipid level measurement, and body composition analysis). Participants diagnosed of diabetes based on a 2-hour OGTT glucose level between 11.1-17.0mml/L (200-300mg/dL) or prediabetes with 2-hour OGTT glucose level between 7.8-11.0 mmol/L (140-199mg/dL), would be followed up with a non-pharmacological lifestyle intervention for one year to assess the effect on their glucose tolerance.

#### Exclusion criteria

Pregnant or breastfeeding women, individuals with pre-existing diabetes (either type 1 or type 2), those with a BMI below 18.5 kg/m², individuals with anemia, and those on cholesterol medications will be excluded from the CarMeR study. These exclusion criteria have been carefully selected to align with the study’s primary objective of ascertaining the prevalence of undiagnosed diabetes within a target population. Pregnant women are excluded due to the possibility of abnormal glucose tolerance precipitated by the pregnancy, and the return to normal glucose tolerance postpartum. Individuals with pre-existing diabetes are excluded to maintain the study’s focus on detecting previously undiagnosed cases, and the exclusion of participants taking high cholesterol medications is a measure taken to address the potential confounding impact of elevated cholesterol levels on diabetes prevalence. In addition, lactating mothers and individuals diagnosed with anemia are excluded because of the blood sample collection. Such participants may be at an increased risk of adverse effects or discomfort associated with the blood drawing process. The exclusion of these categories of people was intended to prioritize participant well-being, conform to the tenets of the Declaration of Helsinki for research on human subjects, and ensure the integrity of the study and the accuracy of its findings.

### Data collection procedures

This is a community-based study, and data collection will be done at the community centers of each community. Data collection is expected to last for five months and the data collection sites for this study include community centers, households, churches and mosques. This will be done after permission is obtained from the gatekeepers in the community for community entry. The gatekeepers include, but not limited to traditional chiefs, opinion leaders, imams, pastors and assembly members. After satisfying all the requirements, ethical clearance for this study has been obtained from the University of Cape Coast (UCC) Institutional Review Board (IRB) with the number UCCIRB/EXT/2022/27. Data will be collected from two or more participants in a single household. For fair representation, participants aged ≥ 25 years will be randomly selected from each household. The purpose of the study, including the benefits and the risks involved will be clearly explained to the participants. After which verbal and written consent will be sought from the prospective participants who have agreed to participate in the study. Written and verbal informed consent will be obtained from each participant for each part of the study, including the survey questionnaires, anthropometric measurements, eye examination and biochemical sample collection.

### Questionnaire/survey questionnaire

The data collection consists of a survey questionnaire, anthropometric measurements, eye examination and collection of biochemical samples. The study will recruit volunteers from communities, markets, churches and schools in the 13 communities in rural, peri-urban and urban Cape Coast.

The WHO-STEPwise approach for identifying and explaining the risk factors for cardiometabolic diseases will be adapted in the data collection process of the CarMeR study. The WHO STEPwise questionnaire is adapted and modified to fit the Ghanaian NCDs context based on previous studies on NCDs in Ghana (45, 46). The first step involves gathering demographic and behavioral risk factor data using a questionnaire; the second step involves the collection of physical measurements; and the third step involves the collection of fasting blood samples for biochemical tests. In addition, the study will collect data on visual outcomes, including retinal changes, visual acuity, contrast sensitivity and visual field. The survey questionnaire consists of seven validated questionnaires that have been adapted to suit Ghanaian culture and the typical Ghanaian lifestyle. The survey questionnaire includes:

1. Perceived Stress Scale (PSS) (47): This questionnaire measures stress levels of participants in the past 1 month.
2. Pittsburgh Sleep Quality Index (PSQI) (48): This questionnaire assesses sleep habits and quality of participants in the past 1 month.
3. International Physical Activity Questionnaire (IPAQ) (49) assesses weekly physical activity levels of the participants.
4. WHO STEPwise Inventory (40): This questionnaire assesses fruits and vegetable intake, smoking and alcohol consumption.
5. Stunkard Figure Rating Scale (50): The Stunkard Figure Rating Scale assesses levels of body type satisfaction using a range of visual representations, starting from very slim (silhouette 1) to the point of significant obesity (silhouette 9).
6. Visual Disability Questionnaire (51): This questionnaire assesses and quantifies the effects of visual impairment on daily activities.
7. A validated questionnaire measuring neighborhood and environmental characteristics.

In addition to these standard questions, data will also be collected on the sociocultural background, demographics, early life experiences, behavioral patterns (hydration habits and substance consumption) environmental exposures, and family history of disease (diabetes, hypertension, diabetes, cancer, asthma). The survey questionnaire is administered by a team of four trained enumerators, and a supervisor who is a senior fellow and cardiometabolic disease researcher with expertise in field surveys. Participants who have consented to participating in the study will be scheduled for a systematic interview. A maximum of ten participants will be engaged each day and the interviews will be conducted right after the first blood sample is collected. The interviews will conducted face-to-face by enumerators with participants in their preferred Ghanaian languages, including Fante, Twi, Ewe, and as well as English for those who are literate and proficient in the English language. The interview for each participant is expected to last approximately 30-45minutes. The interview will be conducted using the Samsung Galaxy Tab E and the data collected from participants will be transmitted to an online server that is managed through the Kobo Collect toolbox application (https://www.kobotoolbox.org). Using the Kobo Collect toolbox, the completeness and quality of the data input are cross-checked by the research assistants and the principal investigator at regular intervals (52, 53).

### Anthropometric measurements

Physical body measurements will be performed using standard equipment validated by international bodies for such protocols. Anthropometric measurements of height, weight, waist circumference, waist-to-hip ratio, and bioimpedance analysis will be performed using the GS6.5B Body Composition Analyzer. Bioimpedance analysis involves the assessment of total body fat, metabolic age, and muscle mass. The device uses these impedance values to calculate various body parameters such as body fat percentage, BMI, muscle mass, hydration status, basal metabolic rate, and metabolic age. Once these measurements are complete, the bioimpedance device displays the results on the screen and produces a printout of the results. Vital blood pressure statistics, including systolic BP, diastolic BP, and pulse rate are measured three times for each participant using the Sinocare BSX 516 Arm-Type Electronic Blood Pressure Monitor. The values of the three measurements are collated and the average is computed.

### Clinical eye examination

The CarMeR study will include a clinical eye examination. The clinical eye examination will be done to assess the status of some selected visual functions and then to screen for visual outcomes that may have been affected by impaired glucose tolerance (IGT). The examination protocol comprises of habitual distant visual acuity measurements, habitual near visual acuity measurements, contrast sensitivity measurement, central visual field measurements, an internal and external eye examination, and assessment of the visual disability index of participants. A qualified optometrist will determine the visual acuity of participants using a Bailey-Lovie or Early Treatment Diabetic Retinopathy Study (ETDRS) visual acuity chart. This is done monocularly at a testing distance of 4 meters with the participants’ usual distance correction. Habitual near visual acuity will also be assessed at 40 cm and recorded in log MAR.

Internal and external eye examination procedures are performed by a qualified optometrist based on the rapid assessment of avoidable blindness (RAAB) methods (54, 55). During this external examination, the eyelids, eyelashes, the eye globe, and anterior segment of the eye are examined. For the internal examination, the main structures to be examined are the lens and the retina. The general retinal area is observed, and the presence or absence of signs of diabetic retinopathy is noted and graded using the International Clinical Diabetic Retinopathy Disease Severity Scale (56). The same is done for the macula and the presence or absence of signs of diabetic macular edema is noted and graded based on the International Clinical Diabetic Macular Edema Disease Severity Scale (56). This is done using the Welch Allyn ophthalmoscope (Welch Allyn, US).

Central Visual Field (CVF) testing is done using the black on white (BOW) Amsler Chart at a distance of 33cm. This is done with the participant’s habitual visual acuity and with near correction, if needed. If any abnormalities such as scotoma, blurry lines, irregular patches, or metamorphopsia are identified, it is categorized as a positive result and the area of visual field is computed. Contrast sensitivity (CS) testing is performed using the Pelli-Robson chart (57). Testing for CS is done at 1 meter where each correctly identified letter receives a score of 0.05 log units, except for the initial triplet. Participants who are deemed to be visually impaired are then administered the visual disability questionnaire (VDQ) adapted from a study on diabetic retinopathy (51). The visual disability score or index is calculated as the mean score within each category, and the sum is calculated for each participant.

Visual impairment classification is determined by visual acuity and visual field, based on the 11th revision of the International Statistical Classification of Diseases, Injuries, and Causes of Death (ICD11) by the World Health Organization (WHO). Mild visual impairment comprises of visual acuity worse than 0.3 log MAR to 0.5 log MAR in the better seeing eye, moderate to severe visual impairment (MSVI) comprises visual acuity worse than 0.5 log MAR to 1.30 log MAR in the better seeing eye, and blindness comprises of visual acuity worse than 1.30 log MAR in the better seeing eye (58).

### Biochemical measurements

#### Fasting blood collection and processing

Eligibility for participating in the OGTT requires participants to fast for at least eight hours, with permission to drink plenty of water from 8pm the night before sample collection. Medical Laboratory Scientists licensed by the Allied Health Profession Council of Ghana will collect the blood specimen using standard venipuncture techniques, with a vacuum blood collection system. Fasting blood specimen of 3ml is collected into one gel separator tube and 2ml each of fasting blood is collected into separate fluoride oxalate tubes for HbA1C and FPG analysis. The blood specimens are kept on ice and transported to the lab for processing and storage. An initial fasting blood glucose test is performed using an Accu-Chek Instant meter and Accu-Chek Instant test strip (Roche, Germany, 2019) to determine glucose tolerance levels and the safety of the participants before subsequent glucose administration for the OGTT procedure. Blood specimen for the other biochemical measurements in this study are also collected in the gel separator tube. These specimens are separated at 3000rpm for 3 minutes and the serum aliquoted into Eppendorf tubes for blood chemistry analysis, immunoassay analysis and storage for reference. For the specimen in the two fluoride oxalate tubes, one is separated and the plasma is aliquoted into Eppendorf tubes and used for fasting blood glucose analysis, whereas the other fluoride oxalate tube sample is used for HbA1c analysis.

To assess the glucose metabolism of participants, an Oral Glucose Tolerance Test (OGTT) is performed as a gold standard diagnostic test. The OGTT assessment uses three time points where blood samples are collected at glucose time of 0, 60 and 120 minutes. The glucose 0’ blood specimen is taken and 82.5g of dextrose monohydrate (equivalent to 75g of anhydrous glucose) is dissolved in 250 ml of drinking water for the participants to drink within 3 minutes. While waiting for their blood to be collected, participants were monitored for any adverse reactions by a trained medical officer. After which blood specimens are taken twice more to assess OGTT glucose level values at 60 minutes and 120 minutes. Each of the time points will be used to diagnose glucose tolerance levels. Prior to the OGTT procedure, spot whole blood glucose analysis is performed to assess Fasting Blood Glucose (FBG). At baseline, blood specimen is also drawn to measure glycated hemoglobin (HbA1C) levels, inflammations (high-sensitivity C-reactive protein [hsCRP], interleukin 6 [IL6]), lipid panels (serum cholesterol, plasma HDL, LDL, TG) and cortisol (a measure of physiologic stress). Specimens for creatinine, liver function tests (AST and ALT), and total protein levels are also taken to measure kidney and renal function. For the OGTT procedure, 10ml of blood is sampled at each time point from each participant, totaling 30ml per participant.

#### Specimen analyses

Spot whole blood glucose analysis is performed using an Accu-Chek Instant meter and Accu-Chek Instant test strip (Roche, Germany, 2019) to determine fasting blood glucose levels. Blood chemistry and Plasma Glucose analysis are conducted using the Mindray BS-240 Blood chemistry analyzer (Mindray, China, August 2022). Glycated hemoglobin (HbA1c), high-sensitivity C-Reactive Protein (hsCRP/CRP), IL6 and Cortisol levels are analyzed using the Wondfo Finecare III Plus Fluorescent Immunoassay analyzer (Wondfo, China, June 2022).

#### Diagnoses

Diagnosis of abnormal glucose tolerance in the CarMeR study will be done using fasting plasma glucose (FPG), HbA1c and OGTT diagnostic tests based on the criteria suggested by the International Experts Committee Report on Diabetes and American Diabetes Association (59, 60). Participants are classified as having prediabetes if they have an FPG level between 5.6mmol/L and 6.9 mmol/L (100mg/dl and 125mg/dl), or an HbA1c level between 5.7% and 6.4% or a 2-hour OGTT result between 140mg/dl and 199mg/dl. Participants are classified as having diabetes if they have an FPG level of 7.0 mmol/L or higher (126mg/dl or higher), or HbA1c level of 6.5% or higher, or a 2-hour OGTT result of 200mg/dl or higher. Additionally, 1-hour post load plasma glucose (PG) will be used to diagnosed hyperglycemia (IH) and diabetes. Participant with a 1-h PG ≥ 155 mg/dL (8.6 mmol/L) are considered to have IH and should be prescribed lifestyle interventions and referred to a diabetes prevention program. Participants with a 1-h PG ≥ 209 mg/dL (11.6 mmol/L) will be considered to have diabetes. Generalized obesity is defined as body mass index (BMI) ≥30 kg/m^2^, and central obesity as a waist circumstance >102 cm in men or >88 cm in women, and a waist-to-hip (WHR of 0.88-0.89 (61, 62). Certified medical laboratory diagnostic scientists will perform all blood sample analysis at the Cardiometabolic Epidemiology Research Laboratory (CERL).

### Recruitment and training of research assistants

The CarMeR study uses two PhD and two MPhil students as research assistants, along with four Laboratory Diagnostic scientists in the study. The four students will be trained on how to establish rapport with the participants, describe the study, collect informed consents with the help of the PI and Co-PI, conduct anthropometric measurements according to the protocol, and administer the survey questionnaire. Laboratory Diagnostic Scientists will be oriented on the specific blood specimen to draw and the corresponding analyses to be performed.

### Enrollment visit

Prior to data collection, recruitment of participants is done by the research assistants (RAs). This is achieved through community engagement via the identified gatekeepers of the specific community. The gatekeepers subsequently organize their community members to engage with the research team. The RAs recruit eligible participants based on the inclusion criteria. The selected participants are assigned codes to anonymize them, and their contacts are collected. Participants who meet the inclusion criteria and agree to participate in the study are asked to sign an informed consent form. Prior to the day of the data collection, participants are called on phone and are reminded of the requirement to fast (8-12 hours), and not ingest any other fluid apart from water. Participants arrive at the study site at 6:30am as communicated to them, and they are asked to rest for a while before their vital signs were measured. Anthropometric measurements are taken, followed by baseline blood samples for FBG, creatinine, liver function tests (AST and ALT), and lipid profiles. After which, a three-point OGTT is performed to assess glucose tolerance. The whole and plasma blood samples are then sent to the CERL at University of Cape Coast, Ghana for analysis and preservation in a cryogenic freezer -40°C. The entire duration of the process is expected to last four hours. Subsequently, participants newly diagnosed with diabetes using a 2-hour glucose level between 11.1-17.0mmol/L (200-299mg/dL) are put into one cohort to complete 12 months lifestyle intervention program and monthly follow-ups. While participants identified with 2-hour glucose ≥ 17.0mmol/L (≥300mg/dL) are referred for medical treatment.

### Data analysis

For the accuracy of data and information, data collected through the Kobo Collect toolbox application will be downloaded into an Excel spreadsheet and screened visually and statistically. Data will be analyzed and interpreted by the principal investigator and his local collaborators and shared back with foreign collaborators. In the attempt to make the findings of the study public, data analysis will be conducted using STATA 16 and SPSS 23. Statistical tools specific and relevant to the research questions and hypotheses of the CarMeR study would be used. To determine the prevalence of undiagnosed diabetes, prediabetes and other associated cardiovascular risks, data on OGTT, FPG and HbA1c will be analyzed using descriptive statistics (percentages and confidence intervals). In order to investigate the differences in phenotype, diabetes, prediabetes, and normoglycemic groups will be compared based on BMI and body composition (waist hip ratio, body fat percentage, skeletal muscle, total body water) using analysis of variance (ANOVA) and t-test. To profile the ocular characteristics and complications of the diabetes and prediabetes groups, a cross-tabulation and a subsequent chi-square test will be done to assess the association between glycemic dysregulation and the presence of ocular complications (diabetic retinopathy, cataract, macular edema). For the purpose of ascertaining whether the predominant etiological factor of undiagnosed diabetes in this populations is insulin resistance or beta-cell failure, a homeostasis model analysis of beta [HOMA-β and HOMA-IR] will be computed and logistic regression will be used to determine the dominant factor. As a way of determining the sensitivity of other diagnostic alternatives (FPG and HbA1c) in comparison to the multi-sampled OGTT, receiver-operated characteristics (ROC curves), including sensitivity and specificity curves, will be used. Finally, to ascertain whether lifestyle changes (food choice, physical activity, weight loss, psychosocial counseling) over a one-year period impacts biomarkers of glucose regulation, a linear mixed model regression analysis, controlled for baseline characteristics will be used. T-tests will be conducted to observe changes in glucose regulation biomarkers.

To manage missing data, imputation and deletion methods would be employed. When the percentage of the missing data is low and not across all observations, the imputation method would be used to estimate the missing data. However, where the number of missing data points is high or the entire data point is missing information, the data point would be deleted during analysis to reduce bias.

### Data management

A casebook will be created for each participant and numbered from UCC0001-UCC1200 based on the sample size of the study. All printed information about the participant is stored in a metal cabinet at the PI’s office with only the PI and the Co-PI having access to the keys. In the case of a future collaboration or requests to use the data collected, participants’ bioinformation will be removed before the data is shared. All sera and plasma would be stored in a freezer at -40C in the CERL for future research and collaboration, with samples being stored for up to 10 years. Leftover blood will be disposed of in two main ways. The first is incineration, where the leftover unused blood samples are sent to the University of Cape Coast Hospital for incineration. The second is decontamination and disposal by adding 0.5% chlorine to the leftover blood for 10 minutes to decontaminate, after which it is deemed safe for the environment, hence, are disposed-off through flushing into covered gutters.

### Ethical considerations

Ethical clearance for the study has been obtained from Institutional Review Board (IRB) of University of Cape Coast (UCC), Ghana. The anticipated potential risks of this are fatigue associated with answering the questionnaire, the discomfort associated with Intra-Venous (IV) insertion, and the drinking of the glucose solution. As a result, a medical practitioner would be present at the study sites at all the times to initially evaluate the participants and oversee IV insertions and blood draws. Qualified and registered phlebotomists/laboratory scientists will perform the IV insertions and blood draws. All discomforts and benefits associated with the entire study would be explained to the participants in their preferred languages, having being assured of confidentiality before written informed consent is obtained for participation in each part of the study (survey, anthropometric body and vital signs measurements, biochemical measures, eye examination). Participants are also made aware of the tests being performed with the collected samples [FBG, glycated hemoglobin (HbA1c), homocysteine, high-sensitive C-reactive protein (hsCRP), lipid panels (serum cholesterol, plasma HDL, LDL, TG), creatinine, liver function tests (AST, ALT), total protein for kidney function] and the amount of blood to be drawn at each time. All participants are counseled on the possibility of being diagnosed with diabetes or prediabetes, how it would affect their lives, and possible solutions. Participants are given the opportunity to withdraw from the study at any time they feel discomfort. Data collected, including participants’ bioinformation are kept in a secure cabinet, accessible only by the PI and the Co-PI. All COVID-19 protocols would be in place to protect both the researchers and participants. All participants who agree to and complete the first part of the study are remunerated.

### Expected outcomes

The CarMeR study is expected to help provide insights on the burden and possible management of diabetes, prediabetes and related cardiometabolic complications in a typical African population. It will ascertain the prevalence of undiagnosed diabetes and prediabetes, as well as explore the relationship between these diseases and cardiovascular risks such as obesity, dietary habits, socioeconomic status, stress, sleep pattern and location. Furthermore, it will provide a nuanced understanding of the phenotypic and biochemical characteristics of the people with diabetes and prediabetes, with a focus on body composition, insulin resistance, and beta-cell function. The study will also describe the ocular characteristics of the participants, in order to identify the ocular complications associated with abnormal glucose tolerance. In the subsequent phase of the study, the efficacy of lifestyle interventions like diet, physical activity, and psychosocial counseling in preventing or reversing diabetes and prediabetes, will be assessed.

## Discussion

Empirical evidence has shown that non-communicable diseases (NCDs), which persist over time, have now emerged as a significant public health challenge worldwide, owing to their alignment with infectious diseases (45, 46, 63). According to the World Health Organization profiles of NCDs, NCDs claim the lives of 41 million individuals annually, accounting for approximately 71% of deaths globally (64). Despite the historically higher prevalence of NCDs in developed nations, a growing concern lies in the fact that they are now common in low-and middle-income countries (LMICs) such as Ghana (65). Cardiometabolic diseases (CMDs) are a group of NCDS, mainly diabetes, coronary heart disease (CHD), and stroke, which are rapidly increasing in prevalence, particularly in LMICs (4, 66–68). Between 2007 and 2017, mortality associated with diabetes, coronary heart disease (CHD), and stroke increased dramatically by 34.7%, 22.3%, and 16.6%, respectively (69). Lifestyle changes remain an essential modifiable factor influencing the risk of mortality in people living with CMDs (29). Previous studies have established a significant relationship between adherence to a healthy lifestyle, including abstinence from smoking, regular physical activity, a balanced diet, and a reduced risk of mortality from CMDs (70–72). It is worth noting that these lifestyle risk factors of severe tobacco use, physical inactivity, and a lack of a balanced diet usually co-occur, making individuals not only susceptible to one type of CMD, but also to all of them. However, there is a paucity of data on the degree to which a combination of lifestyle factors affects the risk of mortality in individuals with CMDs, especially in an African-born population. Given the increasing prevalence of CMDs and consequent increase in mortality, it is essential that there is substantial public health research and promotion of health through culturally sensitive interventions that enable a comprehensive healthy lifestyle (73).

Previous studies (2, 73) have attempted to investigate the risk factors associated with CMDs in Ghana. However, no study has comprehensively explored how demographic characteristics, physical activity and biochemical measures affect the development and progression of CMDs. Certain Ghanaian studies have attempted to explore these possible risk factors, but have done so in isolation and did not account for the influence of other potent factors on the development and progression of CMDs. One study (2) assesses CMDs and their effects on specific organ damage with another (73) mostly focusing on CVDs more than metabolic diseases like diabetes. Understanding the complex interplay of risk factors that lead the genesis and progression of CMDs is essential, especially in the African context. Embracing a multidisciplinary approach that facilitates a more comprehensive evaluation of CMDs development is key to identifying and attempting to remedy the risk factors that predisposes individuals to CMDs (4). Thus, the CarMeR study sought to investigate the risk factors associated with diabetes and one of the most debilitating cardiometabolic diseases in Ghana.

The strength of the CarMeR study is in the incorporation of all three steps of the WHO STEPwise approach (74). As recommended by the World Health Organization, the CarMeR Study utilized all steps to assess risk factors. These steps included the first step being a questionnaire-based assessment, the second step being physical measurements, and the third step being biochemical measures. This reflects the methods of previous studies that have adopted and endorsed the WHO STEPwise approach as an effective NCD surveillance tool (38, 39, 46). The three distinct steps of the WHO STEPwise approach offer a comprehensive approach to understanding risk factors, which is essential for effective intervention and preventive measures.

The CarMeR study is unique because it includes a comprehensive assessment of socio-demographic factors, psychological and behavioral factors, genetic factors and visual factors, complemented by an accurate measure of lifestyle behaviors such as physical activity, and a thorough examination of biochemical parameters of CMDs. With a substantial sample size of 1,200, the utilization of a standard NCD risk factor investigation approach such as the WHO STEPwise approach, and the meticulous application of the biopsychosocial model, the CarMeR study is strategically positioned to determine and subsequently modify the primary risk factors for CMDs within the Ghanaian population.

This study’s conceptual model is based on the biopsychosocial model, which acknowledges the multifactorial nature of CMDs development. Given the marked demographic variations within the Ghanaian population, it is important to investigate how prominent disease determinants such as genetic predisposition, socioeconomic status, family history, occupational influences, early life experiences, lifestyle choices, ethnicity, religion, sleep patterns, and stress levels affect the development of CMDs such as diabetes in an African-born population. The primary objective of this research and future studies from CERL is to provide valuable insights into the prevalence of cardiometabolic diseases and their associated risk factors in Ghana and African-born populations. The findings of this study have the potential to develop and improve interventions for individuals at risk for cardiometabolic diseases within the African context. The CarMeR study promises a positive impact on people affected by cardiometabolic diseases by providing culturally sensitive and adaptable lifestyle interventions. The participants in this study can utilize the results to gain a better understanding of their health status and effectively integrate lifestyle modifications into their daily lives, which can be pivotal in reducing the cardiometabolic disease burden in Ghana and Africa. Furthermore, the prospective results from the CarMeR study hold unlimited potential to uncover previously unexplored pathways in cardiometabolic health, offering opportunities for improved diagnosis and management of cardiometabolic diseases.

The study anticipates some challenges in the execution of this study. The geographical tapestry in the area of interest provides a challenge in reaching the rural communities for the sample collection. To address this, the research team will collaborate with the local gatekeepers to facilitate access and encourage participation. Additionally, cultural dispositions pose challenges, as some community members may prefer not to know their cardiometabolic status, and may not allow their samples to collected. To remedy this, there will be extensive community engagement and campaigns to raise awareness about the importance of the study and its potential health benefits. Another possible challenge could be participants refusing to continue with the study and further sample collection, due to discomfort associated with blood draws. To mitigate this, the study intends to adopt strategies that will minimize discomfort remarkably, such as using experienced phlebotomists, explaining all procedures clearly, and offering support and reassurance to the participants for the entire duration of the study.

Despite these anticipated challenges, it is believed that the robust methods used in the study will facilitate obtaining reliable and valid results. The study results will be released when both phases of the study have been completed.

## Conclusion

The proposed CarMeR study protocol presents a robust method to estimate the burden and determinants of cardiometabolic diseases in Ghana. It utilizes the WHO STEPwise approach to explore and evaluate socio-demographic, psychological, behavioral, genetic, and visual factors. This study holds promise for informing health promotion in Africa due to its large sample size, as well as its comprehensive investigation of lifestyle behaviors and biochemical variables as viable risk factors for CMDs.

The study emphasizes on lifestyle factors being credible risk factors for the development of CMDs like diabetes, and thus will attempt to introduce lifestyle interventions like physical activity, diet, and smoking cessation to improve glycemic levels of participants diagnosed with abnormal glucose tolerance. The study will also stress on the importance of culturally appropriate public health interventions for African-born populations. Thus, the CarMeR study seeks to develop feasible, practical and sustainable interventions to improve health outcomes in people newly diagnosed with the disease.

This study will seek to address an important public health issue in sub-Saharan Africa, using a typical African population in Ghana. The results of its study are expected to contribute to the development of public health policies and guide health promotion and disease prevention efforts. The findings will help expand the existing knowledge on CMDs, especially diabetes, as well as provide better insight into the diagnosis, management, and prevention of CMDs in an African-born population. This research forms a basis for further research and intervention strategies that are targeted at decreasing the impact of CMDs in Ghana and the rest of sub-Saharan Africa.

## Data Availability

The raw data supporting the conclusions of this article will be made available by the authors, without undue reservation.
