# Supplementary material for: Risk factors for cardiometabolic health in Ghana: Cardiometabolic Risks Study Protocol-APTI Project
Source: Front Endocrinol (Lausanne). 2024 Sep 4;15:1337895. doi: 10.3389/fendo.2024.1337895 (PMC11408207; doi:10.3389/fendo.2024.1337895)
Supplement: Supplementary file 1 [file DataSheet1.pdf]

DATA WORKSHEET MODEL-INSTRUMENT
PERSONAL INFORMATION

1. Participant identification (UCC-001) _________
2. Current date ______
3. Last name _________________________
4. First name _______________
5. Date of birth (MM/DD/YY)_________________
6. Cell phone________________/________________
7. Email:______________________________

IDENTIFICATION

8. Participant number (PN)_______
9. Year of visit________
10. Age __________
11. Sex : Man (0) □ Woman (1) □
12. Community_________________
13. Screening site (Put back):
    1. V
    2. W
    3. X
    4. Y
    5. Z

SOCIAL & DIETARY PRACTICES

14. **Marital status:**
    1. Single □
    2. Married □
    3. Widow □
    4. Separated □
    5. Divorced □
15. **Education level (Put back) :**
    1. No education
    2. Basic education (elementary school)
    3. Secondary education (high school)
    4. Tertiary education (college/university)

16. **Profession:**
    1. Fisherfolks □
    2. Farmer □
    3. Government employee □
    4. Business-person □
    5. Market workers □
    6. Student □
    7. Other □ (specify) ………..
    8. None □

17. Household Yearly Income?

18. **Health insurance: 1=Yes / 0=No**

19. **Do you drink alcohol? 1=Yes / 0=No**
   If Yes:

Frequency:
1. Less than once a week □
2. 1 to 6 days a week □
3. Every day □

Type of drink:

1. Local brewery □
2. Packed beer □
3. Packed wine □
4. Liquors □
5. Other (specify) □________________

20. **What source of energy do you use to cook?**

1. Charcoal □
2. Wood □
3. Gas □
4. Electricity □
5. Other □ (specify)___________

21. **In a typical week, on how many days do you eat fruit? (mangoes, oranges, pineapple, banana, etc.)**
    Circle one: 0 1 2 3 4 5 6 7 days
22. **How many servings of fruit do you eat on one of those days? _________** servings/day

23. **In a typical week, on how many days do you eat vegetables? (spinach, tomatoes, lettuce, onion, etc.)**
    Circle one: 0 1 2 3 4 5 6 7 days

24. **How many servings of vegetables do you eat on one of those days? _________** servings/day

25. **How many times do you consume energy-dense food in a day? (rice, fufu, kenke, banku, re-red, etc.)**

    0 = never
    1 = once
    2 = twice
    3 = thrice or more

26. **How many servings of these energy-dense foods do you consume at one sitting?**
    _______servings/meal

27. What type of oil or fat is most often used for meal preparation in your household?

- Vegetable oil
- Palm oil
- Butter or ghee
- Margarine
- Other
- None
- Don't know

28. How often do you eat in restaurants and pubs?

1. Less than once a week □
2. 1 to 3 days a week □
3. 4 to 6 days a week □
4. Everyday □

29. What kind of condiments do you add to your food?

1. Mayonnaise □
2. Salt □
3. Chili pepper □
4. Butter □
5. Other □ (specify)_______________
6. None □

30. Do you drink soda? 1=Yes/ 0= No

If yes

How often do you drink soda (fanta, malt, sobolo, coca-cola)?

0 = Less than once a week □
1 = 1 to 3 days a week □
2 = 4 to 6 days a week □
3 = Everyday □

31. Do you drink energy drinks? 1 = Yes / 0 = No

If yes

How often do you drink energy drinks?

0 = Less than once a week □
1 = 1 to 3 days a week □
2 = 4 to 6 days a week □
3 = Everyday □

32. Do you drink tea? 1=Yes/ 0= No

If yes

- **How often do you drink tea?**

  0 = Less than once a week □
  1 = 1 to 3 days a week □
  2 = 4 to 6 days a week □
  3 = Everyday □

- **How many teaspoons of sugar/honey do you add to your tea?**

1. 1 □
2. 2 □
3. 3 □
4. 4 □
5. > 4 □
6. None □

33. Do you drink porridge (koko)? 1=Yes/ 0= No
If yes

- **How often do you drink koko?**

5. Less than once a week □
6. 1 to 3 days a week □
7. 4 to 6 days a week □
8. Everyday □

- **How many teaspoons of sugar do you add to your koko?**

1. 1 □
2. 2 □
3. 3 □
4. 4 □
5. >4 □
6. None □

34. Do you smoke?
0= No
1= Currently smoke
2= Passive smoking (close contact who smoke)
3= Smoked in the past
If Yes :
Number of cigarettes per day…….
35. Do you use any other kind of tobacco: 1=Yes / 0=No
If yes,
1. Shisha (hookah) □
2. Chewing tobacco □
3. Pipe □

4. Other □……………….

PAST MEDICAL HISTORY

36. Has anyone in your family has diabetes? 1=Yes/ 0= No

If yes:

1. Mother □
2. Father □
3. Brother □
4. Sister □
5. Grandmother
6. Grandfather

37. Has a health professional told you, you have high blood pressure (Hypertension)? 1=Yes / 0=No

38. Are you on blood pressure medication? 1=Yes / 0=No

39. Do you have any other medical condition? 1=Yes / 0=No

If yes:

1. Heart failure □
2. Blood disorder (anemia) □
3. Liver disease □
4. Kidney disease □
5. Other (specify)□______

SLEEP QUALITY

1. What time do you go to bed at night?

   Time _ _ : _ _ (hours)

2. How long does it take you to fall asleep? (sleep latency)

   Time _____ (minutes)

3. What time do you wake up in the morning?

   Time _ _ : _ _

4. How many hours of actual sleep do you get each night? (this may be different from the number of hours you spend in bed)
   1. <5 hours
   2. 5-6 hours
   3. 6-7 hours
   4. 7-8 hours
   5. >8 hours

Sleep disturbances: During the past month, how often did you have trouble sleeping in the night because:

5. You cannot fall asleep within 30 minutes when getting to the bed?
   0 = Not during the past month
   1 = less than once a week
   2 = once or twice a week
   3 = three or more times a week
6. You have to wake up to use the bathroom?
   0 = Not during the past month
   1 = less than once a week
   2 = once or twice a week
   3 = three or more times a week
7. You wake up suddenly, in the middle of the night?
   0 = Not during the past month
   1 = less than once a week
   2 = once or twice a week
   3 = three or more times a week
8. You had a bad dream?
   0 = Not during the past month
   1 = less than once a week
   2 = once or twice a week
   3 = three or more times a week
9. You had pain?
   0 = Not during the past month
   1 = less than once a week
   2 = once or twice a week
   3 = three or more times a week
10. You felt too cold?
    0 = Not during the past month
    1 = less than once a week
    2 = once or twice a week
    3 = three or more times a week
11. You felt too hot?
    0 = Not during the past month
    1 = less than once a week
    2 = once or twice a week
    3 = three or more times a week
12. You coughed loudly?
    0 = Not during the past month
    1 = less than once a week

2 = once or twice a week
3 = three or more times a week

13. During the past month, how often did you use sleep medications (prescription or OTC)?
    0 = Not during the past month
    1 = less than once a week
    2 = once or twice a week
    3 = three or more times a week
14. How would you rate your overall sleep quality?
    1. Very good
    2. Fairly good
    3. Fairly bad
    4. Very bad

Final PSQI Score: ____________________

COHEN PERCEIVED STRESS SCALE (PSS10)

1. In the past month, how often have you been upset because of something that happened unexpectedly?

2. In the past month, how often have you felt unable to control the important things in your life?

3. In the past month, how often have you felt nervous our stressed?

4. In the past month, how often have you found that you could not cope with all the things you had to do?

5. In the past month, how often have you been angry because of things that had happened that were out of your control?

6. In the past month, how often have you felt that difficulties were piling up so high that you could not overcome them?

0 = Never
1 = Almost never
2 = Sometimes
3 = Fairly often
4 = Very often

7. In the past month, how often have you felt confident about your ability to handle personal problems?

8. In the past month, how often have you felt that things were going your way?

9. In the past month, how often have you been able to control irritations in your life?

10. In the past month, how often have you felt that you were on top of things?

0 = Very often
1 = fairly often
2 = sometimes
3 = almost never
4 = never

Final PSS Score: _______________

LIFESTYLE / PHYSICAL ACTIVITY QUESTIONS

15. What is your usual means of transportation to and from places?

1. Walking □
2. Bicycle □
3. Motorbike □
4. Bus □
5. Private Car □
6. Mixture of the above □

16. On average how much time do you spend walking every day?

1. Less than 1 hour □
2. 1 to 2 hours □
3. 3 to 4 hours □
4. 5 to 6 hours □
5. More than 6 hours □

17. What kind of work do you do with regards to physical activity?

1. Mostly sitting (Computer based, shop keepers, drivers) □
2. Sitting and frequently walking (Healthcare, teachers, …) □
3. Mostly walking around (security personnel, mail delivery,…) □
4. Physical labor (fishing, farming, construction, mechanics,…) □

18. Do you do sports? 1=Yes / 0=No

If yes, How often do you do sports?

1. Less than once a week □
2. 1 to 3 days a week □
3. 4 to 6 days a week □
4. Every day □

- **What kind of sports do you do?**

1. Jogging, swimming, cycling □
2. Ball/field/net games (Football, basketball, volleyball, tennis, badminton, …) □
3. Dance exercises (gym, jump rope, aerobics,...) □
4. Weight lifting □
5. Multiple category □

- **How much time do you spend doing sports?**

1. Less than 30 min □
2. 30 min to 1 hour □
3. 1 to 2 hours □
4. More than 2 hours □

INTERNATIONAL PHYSICAL ACTIVITY QUESTIONNAIRE

We are interested in finding out about the kinds of physical activities that people do as part of their everyday lives. The questions will ask you about the time you spent being physically active in the **last 7 days**. Please answer each question even if you do not consider yourself to be an active person. Please think about the activities you do at work, as part of your house and yard work, to get from place to place, and in your spare time for recreation, exercise or sport.
Think about all the **vigorous** activities that you did in the **last 7 days**. **Vigorous** physical activities refer to activities that take hard physical effort and make you breathe much harder than normal. Think *only* about those physical activities that you did for at least 10 minutes at a time.

1. During the **last 7 days**, on how many days did you do **vigorous** physical activities like heavy lifting, digging, aerobics, jogging, gym activities, or fast bicycling?
   _____ Days per week
   _____ No vigorous physical activities ***Skip to question 3***

2. How much time did you usually spend doing **vigorous** physical activities on one of those days?
   _____ Hours per day
   _____ Minutes per day
   _____ Don’t know/Not sure

8.0 METs X ______ min/day X ______ days/week=_______MET min/week

Think about all the **moderate** activities that you did in the **last 7 days**. **Moderate** activities refer to activities that take moderate physical effort and make you breathe somewhat harder than normal. Think only about those physical activities that you did for at least 10 minutes at a time.

3. During the **last 7 days**, on how many days did you do **moderate** physical activities like carrying light loads, bicycling at a regular pace, or doubles tennis? Do not include walking.
   _____ Days per week
   _____ No moderate physical activities ***Skip to question***

4. How much time did you usually spend doing **moderate** physical activities on one of those days?
   _____ Hours per day
   _____ Minutes per day
   _____ Don’t know/Not sure

6.0 METs X ______ min/day X ______ days/week=_______MET min/week

Think about the time you spent **walking** in the **last 7 days**. This includes at work and at home, walking to travel from place to place, and any other walking that you have done solely for recreation, sport, exercise, or leisure.

5. During the **last 7 days**, on how many days did you **walk** for at least 10 minutes at a time?
   _____ Days per week
   _____ No walking ***Skip to question 7***

6. How much time did you usually spend **walking** on one of those days?
   _____ Hours per day
   _____ Minutes per day
   _____ Don't know/Not sure

3.3 METs X ______ min/day X ______ days/week=_______MET min/week

The last question is about the time you spent **sitting** on weekdays during the **last 7 days**. Include time spent at work, at home, while doing course work and during leisure time. This may include time spent sitting at a desk, visiting friends, reading, or sitting or lying down to watch television.

7. During the **last 7 days**, how much time did you spend **sitting** on a **week day**?
   _____ Hours per day
   _____ Minutes per day
   _____ Don't know/Not sure

8. **Continuous score for physical activity:__________ MET min/week**
   (Total MET min/wk)

9. **Categorical score for physical activity: (Circle one)**

   1=Low 2=Moderate 3=High

*reference: Baldi S., et al. (2013). Influence of Apolipoproteins on the Association between Lipids and Insulin Sensitivity. *Diabetes Care,* 36(12), 4125-4131.

1. Which best describes your activities of your daily life? _____

   1= **Inactive** - No regular physical activity with a sit down job, including hospitalized patients
   2= **Light** – No organized physical activity during leisure time with three to four hours of walking or standing per day
   3= **Moderate** – Sporadically involved in recreational activities such as weekend golf or tennis, occasional jogging, swimming, or cycling
   4= **Heavy** – Consistent job activities of lifting or stair climbing or participating in recreational/fitness activities such as jogging, swimming or cycling at least 3 times per week for 30 to 60 minutes per session
   5= **Vigorous** – Participation in extensive physical activity for 60 minutes or more minutes at least 4 times per week

2. Do you work on the computer every day? (1=Yes/2=No) _____

3. Do you watch TV every day? (1=Yes/2=No) _____

4. How many hours per day do you watch TV or spend on the computer, combined? _____

0 = Zero hours
1 = 0.01 to three hours
2 = 3.01 to six hours
3 = 6.01 to nine hours
4 = More than nine

Stunkard Scale - Body Size

1. Choose your ideal figure for yourself from the following pictures.

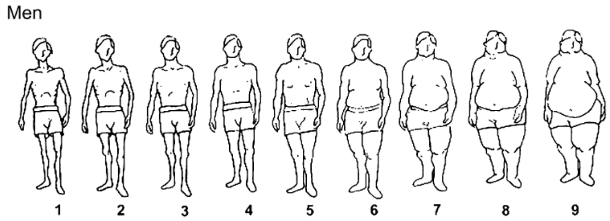

2. Choose the figure that reflects how you think you look.

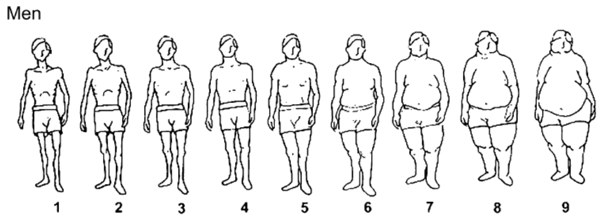

*reference: Lynch E., et al. (2008). The Relationship Between Body Size Perception and Change in Body Mass Index Over 13 Years. *American Journal of Epidemiology*, 169(7), 857-866.

Stunkard Scale - Body Size

1. Choose your ideal figure for yourself from the following pictures.

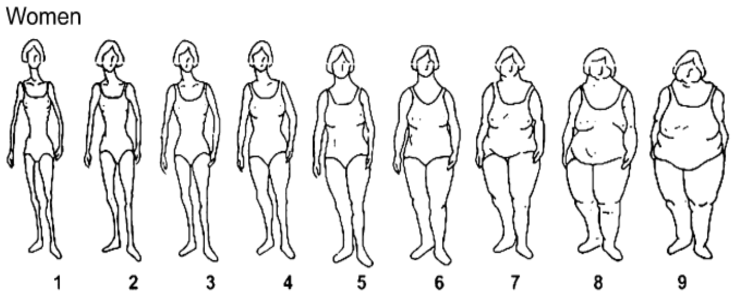

2. Choose the figure that reflects how you think you look.

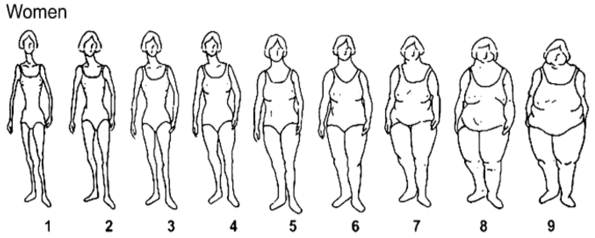

Reference: Lynch E., et al. (2008). The Relationship Between Body Size Perception and Change in Body Mass Index Over 13 Years. *American Journal of Epidemiology*, 169(7), 857-866.

EARLY LIFE EXPERIENCES

Were you malnourished as a child?

0 = No
1 = Underfed
2 = Well-fed
3 = Overfed

Were you overweight as a child (obolo)? **0 = No / 1 = Yes**

How would you describe the socioeconomic status of parents when you were a child?

0 = poor
1 = middle class
2 = rich / upper class

PHYSICAL EXAM AND LABORATORY INVESTIGATIONS

19. Anthropometrics

Weight (Kg):
Height (m):
BMI:
Waist Circumference (WC) in cm: WC 1___ WC 2____ WC 3___ Avg WC (comp gen)_____
Hip circumference (HC) in cm: HC 1___ HC 2____ HC 3___ Avg HC (comp gen)_____
WHR (comp gen)__________
Total body fat: __________
Metabolic age: ___________

20. Blood pressure

Systolic blood pressure (mmHg):_________
Diastolic blood pressure (mmHg):_________
Pulse rate (BPM): ___________

21. Laboratory investigations

1. Glucose at – 15 min_____
2. Glucose at 0 min
3. Glucose at 120 min
4. Hemoglobin A1C____%
    a. HPLC Method+_____

5. Glycated albumin___%
6. Total albumin (g/L)
7. Plasma insulin at – 15 min_____
8. Plasma insulin at 0 min (pmol/L)___
9. Plasma insulin at 120 min (pmol/L)___
10. Total cholesterol (mmol/L)_____
11. HDL cholesterol (mmol/L)_____
12. Triglycerides (mmol/L)____-
13. LDL cholesterol_____
14. Homocysteine _____
15. High-sensitivity C-reactive protein ______
16. Fibrinogen _______
17. Hemoglobin: ________g/dL
18. Hematocrit_______%
19. Hemoglobin electrophoresis:
    a. HbA
    b. HbAA
    c. HbA2
    d. HbS
    e. HbC
    f. Other

22. Result of OGTT

1. Normal glucose tolerance
2. Prediabetes if yes: impaired fasting glucose , glucose intolerance, both
3. Diabetes
